# Attenuation of replication by a 29 nucleotide deletion in SARS-coronavirus acquired during the early stages of human-to-human transmission

**DOI:** 10.1038/s41598-018-33487-8

**Published:** 2018-10-11

**Authors:** Doreen Muth, Victor Max Corman, Hanna Roth, Tabea Binger, Ronald Dijkman, Lina Theresa Gottula, Florian Gloza-Rausch, Andrea Balboni, Mara Battilani, Danijela Rihtarič, Ivan Toplak, Ramón Seage Ameneiros, Alexander Pfeifer, Volker Thiel, Jan Felix Drexler, Marcel Alexander Müller, Christian Drosten

**Affiliations:** 1 Charité-Universitätsmedizin Berlin, corporate member of Freie Universität Berlin, Humboldt-Universität zu Berlin, and Berlin Institute of Health, Institute of Virology, Charitéplatz 1, 10117 Berlin, Germany; 2 German Center for Infection Research (DZIF), Berlin, Germany; 3 Institute of Virology, University of Bonn Medical Centre, Sigmund-Freud-Str. 25, 53127 Bonn, Germany; 4 Federal Department of Home Affairs, Institute of Virology and Immunology IVI, Bern and Mittelhäusern, Sensemattstrasse 293, 3147 Mittelhäusern, Switzerland; 5 Department of Infectious Diseases and Pathobiology, Vetsuisse Faculty, University of Bern, Länggassstrasse 122, 3012 Bern, Switzerland; 6 Noctalis, Centre for Bat Protection and Information, Oberbergstraße 27, 23795 Bad Segeberg, Germany; 7 Dipartimento di Scienze Mediche Veterinarie, Facoltà di Medicina Veterinaria, Alma Mater Studiorum-Università di Bologna, Via Tolara di Sopra 50, 40064 Ozzano Emilia, (BO) Italy; 8 Virology Unit, Institute of Microbiology and Parasitology, Veterinary Faculty, University of Ljubljana, Gerbičeva 60, 1000 Ljubljana, Slovenia; 9 Institute of Evolutionary Ecology and Conservation Genomics, University of Ulm, Albert-Einstein Allee 11, 89069 Ulm, Germany; 10 Group Morcegos de Galicia, Drosera Society, Pdo. Magdalena, G-2, 2° esq, 15320 As Pontes, Spain; 11 Institute for Pharmacology and Toxicology, University of Bonn, Sigmund-Freud-Str. 25, 53127 Bonn, Germany

## Abstract

A 29 nucleotide deletion in open reading frame 8 (ORF8) is the most obvious genetic change in severe acute respiratory syndrome coronavirus (SARS-CoV) during its emergence in humans. In spite of intense study, it remains unclear whether the deletion actually reflects adaptation to humans. Here we engineered full, partially deleted (−29 nt), and fully deleted ORF8 into a SARS-CoV infectious cDNA clone, strain Frankfurt-1. Replication of the resulting viruses was compared in primate cell cultures as well as *Rhinolophus* bat cells made permissive for SARS-CoV replication by lentiviral transduction of the human angiotensin-converting enzyme 2 receptor. Cells from cotton rat, goat, and sheep provided control scenarios that represent host systems in which SARS-CoV is neither endemic nor epidemic. Independent of the cell system, the truncation of ORF8 (29 nt deletion) decreased replication up to 23-fold. The effect was independent of the type I interferon response. The 29 nt deletion in SARS-CoV is a deleterious mutation acquired along the initial human-to-human transmission chain. The resulting loss of fitness may be due to a founder effect, which has rarely been documented in processes of viral emergence. These results have important implications for the retrospective assessment of the threat posed by SARS.

## Introduction

Emerging zoonotic viruses such as Severe Acute Respiratory Syndrome coronavirus (SARS-CoV) are a considerable concern for public health^1^. Bats of the genus *Rhinolophus* are the *bona-fide* animal reservoir for SARS- and SARS-related CoV (SARSr-CoV)^2^. Epidemics may emerge from wild animal reservoirs in which a plethora of viral variants exists. After initial cross-host infection, the occurrence of positive selection with adaptive changes is thought to be essential for viral emergence^3,4^. The confirmation of phenotypic changes acquired in the process of adaptation requires virus isolation, which is not always possible. In the case of SARS-CoV, isolation of virus strains from the bat reservoir has widely failed^5^, except in singular instances involving bat-borne viruses whose spike protein showed an exceptionally high amino acid identity with the epidemic strain^6–8^. Additional reasons for low isolation success include the lack of appropriate cell culture systems as well as low virus concentrations and often inappropriate storage conditions for samples from field investigations. Studies of phenotypic properties of reservoir-borne viruses can be facilitated by the reconstruction of genetic traits through reverse genetics.

In the present study we reconstructed variant SARS-CoVs carrying different forms of open reading frame (ORF) 8, an accessory gene in the SARS-CoV genome that is among the most variable genes in bat-associated SARSr-CoV. ORF8 is also one of the most relevant genes when studying potential viral adaptation to humans, as the ORF8 coding sequence has undergone gradual deletion during the human epidemic. Early epidemic SARS-CoVs contained a full ORF8 that is also present in the genomes of almost all bat- and carnivore-associated precursor viruses^9–12^. A 29 nucleotide (nt) deletion within ORF8 occurred in all strains involved in the middle and late phase of the human epidemic^9,10^. Subtotal or total deletions of up to 415 nts occurred within and around the ORF8 region in SARS-CoV strains circulating in the very late phase of the epidemic. We have previously found that a European SARS-related CoV carried by rhinolophid bats in Bulgaria did not contain any ORF8 or similar gene^13^ while virtually all SARS-related CoV from Asia possess a single, continuous ORF8^8,14,15^.

The 29 nt deletion in ORF8 was the most obvious genetic change during human-to-human transmission of SARS, causing the expression of truncated gene products termed ORF8a and ORF8b. It has been widely hypothesized that the truncated products led to a modulation of pathogenicity or replication that favored adaptation of SARS-CoV to humans^9,11,12^. For instance, it was found that replication of SARS-CoV is increased in cells that overexpress the protein encoded by ORF8a^16^. In contrast, the same protein led to an attenuation of replication when engineered into recombinant infectious bronchitis virus (IBV)^17^. The 8b protein, when overexpressed, was found to induce apoptosis and to be involved in cellular degradation of the viral envelope protein during SARS-CoV infection^16,18,19^. When engineered into an IBV infectious clone, protein 8b inhibited the induction of interferon (IFN) as triggered by poly(I:C)-stimulation and thus enhanced IBV replication. This effect was found to involve binding of ubiquitin and interference with induction of the IFN type 1 promoter via IRF3^17^. Another study found that the full 122 amino acid protein encoded by ORF8 induces ATF6-dependent transcription, which triggers the expression of chaperones and leads to a general attenuation of the protein translation level, thus modifying the unfolded protein response^20^. Loss of ORF8’s original cellular localization in the endoplasmatic reticulum would have ablated this function in human viruses of the middle and late epidemic phases^20,21^. However, the effect that this change had on SARS-CoV replication level remains unclear. Studies of SARS-CoV replication in experimental mice have not provided indications as to essential functions of ORF8a or 8b^22^. However, these studies involved high inoculated virus doses and utilized a mouse model that may not reflect relative changes of replication in transition from bats to humans. In sum, the available data leave it unclear whether the changes that occurred in ORF8 led to a modification of viral replication when comparing bat versus human hosts, and specifically, whether the deletion of 29 nt involved an increase of replication level in human cells as often implicated. Genome deletions have also been observed in MERS-CoV, and also here it was speculated that deletion may reflect adaptation to the human host or the release of selective pressure exerted exclusively in the zoonotic reservoir^23,24^.

In the present study on SARS-CoV, we first followed up on our initial finding of the absence of ORF8 in genomes of SARSr-CoV from European bats, and sequenced rhinolophid bat-associated SARSr-CoV from four countries across Europe (Spain, Bulgaria, Italy, Slovenia). We then studied the influence of ORF8 on replication in the context of a full replicating SARS-CoV genome, based on a host transition scenario represented by cell cultures. We constructed recombinant viruses with full ORF8, truncated ORF8 (29 nt deletion), as well as completely deleted ORF8. Replication was compared in primate cell lines (VeroFM, MA104), a novel cell line generated from the lung of a rhinolophid bat, three additional non-chiropteran cell lines, as well as human airway epithelial cultures. We find that the 29 nt deletion conferred an attenuation of replication level irrespective of the host system studied.

## Results

### Bats in Europe carry SARS-related CoV that lack ORF8

We have previously described the detection of SARS-related CoV in *Rhinolophus* in Bulgaria^13^. The full-length genome of the virus sequenced in that study contained no ORF8. To further investigate the presence of ORF8 in SARS-related CoV in Europe, we analyzed fecal samples from rhinolophid bats in Spain, Bulgaria, Italy, as well as Slovenia. All five *Rhinolophus* species commonly encountered in Europe were tested positive for coronavirus RNA (Table 1). However, none of 92 geographically and phylogenetically representative viruses provided evidence for the presence of an ORF8 gene. 19 of these viruses were fully sequenced, confirming absence of ORF8 at orthotopic or heterotopic genome positions.

**Table 1:** Bat specimens included in this study and confirmation of absence of ORF8.

Country	N individuals tested	Rhinolophid species and number of individuals (n) yielding positive RT-PCR	Number of RT-PCR results indicating absence of ORF8
Bulgaria	506	*R. euryale* (n = 44) *R. blasii* (n = 11) *R. ferrumequinum* (n = 1) *R. mehelyi* (n = 4)	44 11 1 4
Italy	45	*R. ferrumequinum* (n = 6)	6
Slovenia^53^	36	*R. hipposideros* (n = 5)	5
Spain	285	*R. hipposideros* (n = 21)	21
Total	872	All 5 species known in Europe	92 (100%)

Absence of ORF8 was determined by RT-PCR using reverse primers F29260R (TTTGTATGCGTCAATGTGCTTG) for reverse transcription and F28182R (GGGTCCACCAAATGTAATGCGG) and forward primer F27626F (GAGAAAGACAGAATGAATGAGC) for PCR.

### Generation of recombinant SARS-CoV encoding ORF8 variants

**Figure 1:**
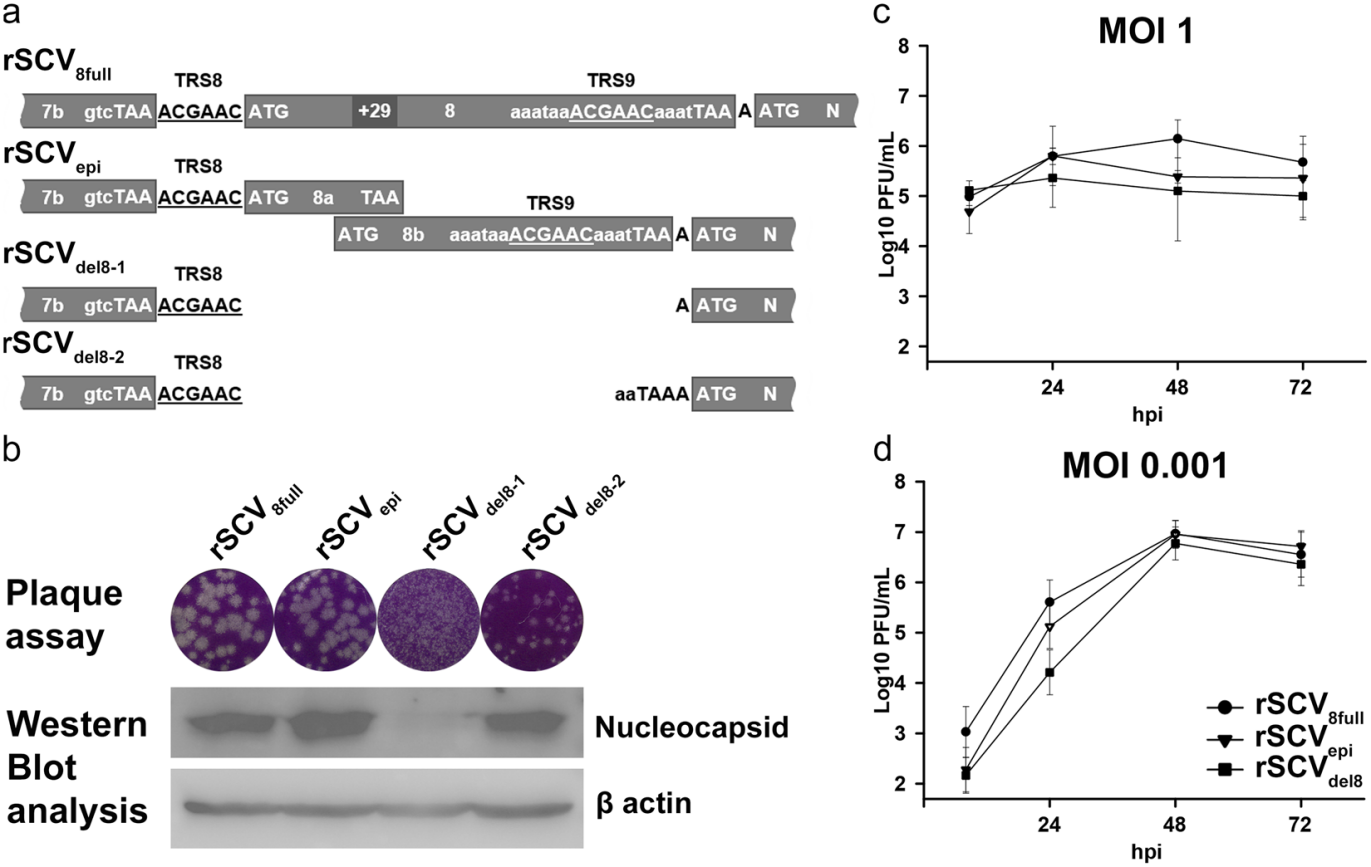
Generation and evaluation of ORF8 variant recombinant SARS-CoV. Variants of the open reading frame 8 (ORF8) were designed in accordance to their appearance in nature. (**a**) rSCV_8full_ represents a single ORF8 as found in reservoir bats and amplification hosts in China, as well as in the early phase of the SARS epidemic. The middle phase of the epidemic was dominated by a virus variant carrying a 29 nt deletion resulting in the disruption of ORF8 in 2 reading frames, 8a and 8b, as seen in rSCV_epi_. The absence of ORF8 is a genomic feature of reservoir bats in Europe and the epidemic virus in the late phase. Two deletion variants were constructed. Variant 1, rSCV_del8–1_, perfectly misses ORF8. In variant 2, rSCV_del8–2_, ORF8 is replaced by the short substitutional sequence AATAA in accordance to the upstream region of the nucleocapsid gene of the European SARS-related bat-CoV BtCoV/BM48–31/Rhi bla/Bulgaria/2008 (NC_014470). (**b**) Plaque morphology of rSCV_8full_ and rSCV_epi_ were very similar, while rSCV_del8–1_ produced only diffuse and rSCV_del8–2_ reduced plaques. Western Blot analysis revealed that only rSCV_8full_, rSCV_epi_, and rSCV_del8–2_ infected cells expressed the nucleocapsid protein, while none could be detected in cells infected with rSCV_del8–1_. Detection of β actin served as loading control. Virus replication of the three ORF8 variants, (rSCV_del8_ = rSCV_del8–2_) was monitored by plaque titration after infection of VeroFM cells at two different multiplicities of infection, 1 (**c**) and 0.001 (**d**). Virus growth was determined in at least 3 independent experiments in triplicates. Shown is one representative experiment. Error bars represent standard deviation of the mean.

To understand the consequences of the absence of ORF8, recombinant SARS-CoV (rSCV) were generated as shown in Fig. 1a. The variant termed rSCV_8full_ contained a complete ORF8 as encountered in bats, civet cats, as well as in early human cases of SARS, thus resembling the pre-epidemic and starting phase of the SARS outbreak. The variant termed rSCV_epi_ corresponds to isolates from the main phase (also referred to as middle and late phases) of the epidemic containing a 29 nt deletion that introduces a ribosomal frameshift separating ORF8 into ORF8a and ORF8b. Two alternative virus variants without ORF8 were constructed. Variant 1, rSCV_del8–1_, had ORF8 removed without any replacement. Variant 2, rSCV_del8–2_, had ORF8 replaced by a 5 nt spacer sequence (AATAA) which occurs instead of ORF8 between ORF7b and the nucleocapsid gene in a natural SARS-related CoV variant from European rhinolophid bats^13^.

Infectious viruses were rescued from cDNA as described^25^. 24 h after co-electroporation of *in-vitro* transcribed RNA and nucleocapsid subgenomic RNA, parts of the cell culture supernatant were transferred to VeroFM cells and virus replication was monitored by real-time RT-PCR. 72 h post infection (hpi), rSCV_8full_, rSCV_epi_ and rSCV_del8–2_ gave evidence of replication by real-time RT-PCR, while rSCV_del8–1_ RNA increased more slowly, reaching a plateau level only by 96 hpi.

All variants were plaque-quantified. During this process, differences in plaque morphology were noted. While rSCV_8full_ and rSCV_epi_ yielded plaques of similar sizes, rSCV_del8–2_ showed smaller plaque size and rSCV_del8–1_ generated no distinct plaques (Fig. 1b).

The absence of plaques in rSCV_del8–1_ could point to a replication defect caused by the loss of essential gene functions, including the nucleocapsid gene whose expression is indispensable for virus replication^26^. Modifications of ORF8 affect the upstream context of the transcription regulatory sequence (TRS) of the nucleocapsid gene^27^. Western blot analyses showed that rSCV_8full_, rSCV_epi_, as well as rSCV_del8–2_ expressed similar amounts of the nucleocapsid protein, while no expression was seen in cells infected with rSCV_del8–1_ (Fig. 1b). Subsequent experiments were therefore carried out using variant 2, which is hereafter referred to as rSCV_del8_.

### Integrity of ORF8 facilitates the replication of recombinant SARS-CoV

Replication of rSCV_8full_, rSCV_epi_, and rSCV_del8_ were tested in VeroFM cells at high and low multiplicity of infection (MOI) (1 and 0.001 plaque forming unit (PFU)/cell). Supernatants were harvested at 8, 24, 48, and 72 hpi and titered in VeroFM cells (Fig. 1c, d). At MOI = 1, 30–40% of the cells were already lysed by 24 hpi, precluding meaningful comparisons of viruses (Fig. 1c). At MOI = 0.001, replication was in the exponential phase by 24 hpi (Fig. 1d). rSCV_8full_ grew significantly more efficiently than both other variants at 24 hpi (p < 0.05 and p < 0.001 comparing to rSCV_epi_ and rSCV_del8_, respectively, using *t*-Test). The fully deleted variant rSCV_del8_ replicated significantly less efficiently than rSCV_epi_ at 24 hpi (p = 0.002, *t*-Test). By 48 hpi, replication of all variants reached plateau levels.

### The replication facilitating effect of ORF8 is IFN independent

Due to the essential role of the type I IFN system in the restriction of virus infection, it was tested whether the ORF8-dependent replication phenotype might be linked to the type I IFN response. MA104 monkey kidney cells were used because they are competent for IFN induction and signaling^28^. VeroFM cells would only reflect differences in IFN signaling, due to their defect in IFN beta gene expression^29^. According to previous results, virus infections with all three variants were carried out at MOI = 0.001 and virus progeny was measured at 24 hpi by plaque assay.

**Figure 2:**
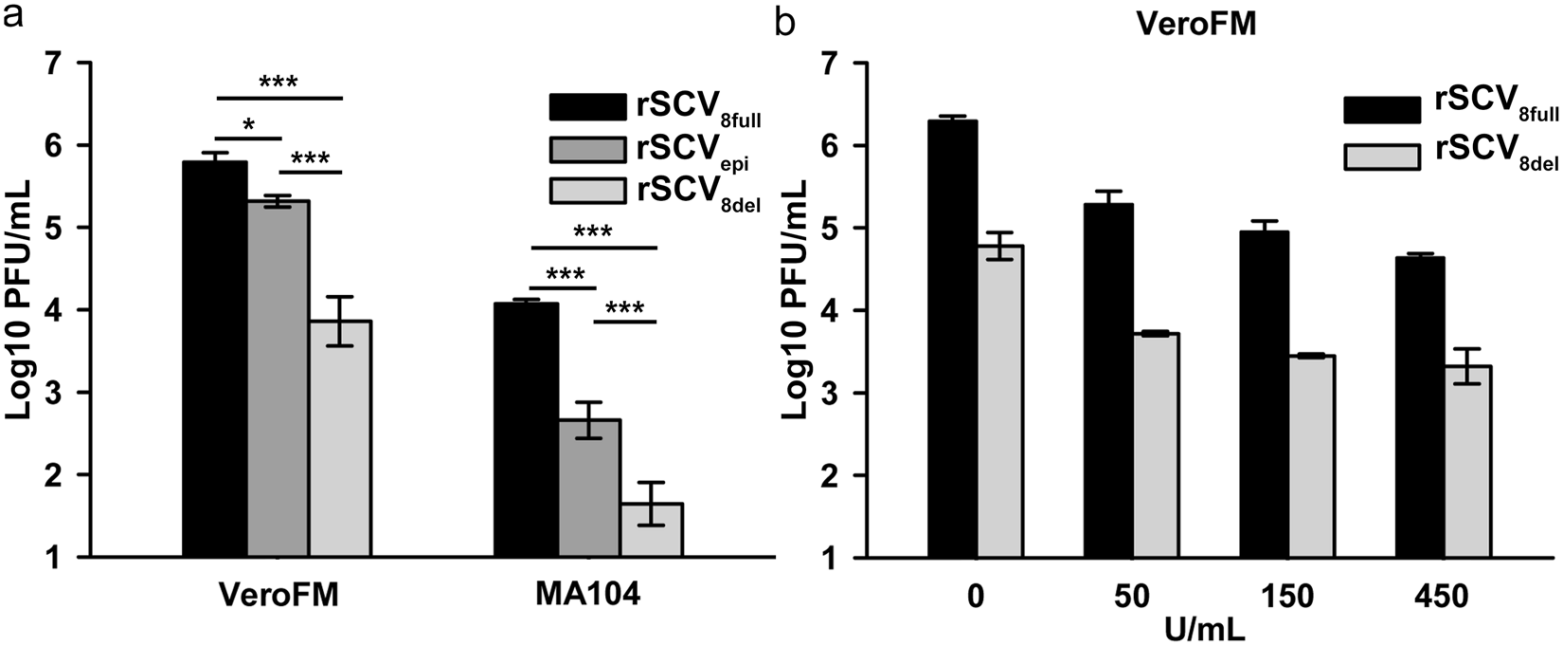
Replication of ORF8 variants in primate cell cultures with and without IFN pre-treatment. (**a**) VeroFM and MA104 cells were infected with three ORF8 variant viruses at MOI = 0.001. Supernatants were harvested at 24 hpi and virus titers determined by plaque titration. The experiment was done in triplicates. Significance of replication differences between virus variants was determined by *t-Test* (***p < 0.001, *p < 0.05). (**b**) VeroFM cells were incubated with universal type I IFN at indicated concentrations 16 h prior to infection with rSCV_8full_ and rSCV_del8_ at MOI = 0.001. At 24 hpi supernatants were plaque titered. Experiments were done at least twice in triplicates. Shown is one representative experiment each. Error bars represent standard deviation of the mean.

All viruses generally replicated more efficiently in VeroFM cells than MA104 cells. However, relative differences between strains were very similar in both cell lines, suggesting that IFN beta gene induction that is only functional in MA104 cells may not play a significant role for the observed phenotypes (Fig. 2a).

To test the potential influence of the ORF8 gene product on IFN signaling, virus growth after external addition of IFN was compared. To this end, VeroFM cells were incubated with indicated concentrations of universal type I IFN alpha prior to infection with rSCV_8full_ and rSCV_8del_. At 24 h p.i., rSCV_8full_ grew to titers 30 times higher than rSCV_del8_ (Fig. 2b). If cells were treated with IFN prior to infection the overall replication of both viruses decreased in a dose-dependent manner. However, the replication difference between both viruses remained constant. This suggests that ORF8 has a SARS-CoV-specific replication-enhancing effect that is independent of IFN antagonism.

### ORF8 improves replication of recombinant SARS-CoV in a reservoir bat cell line

**Figure 3:**
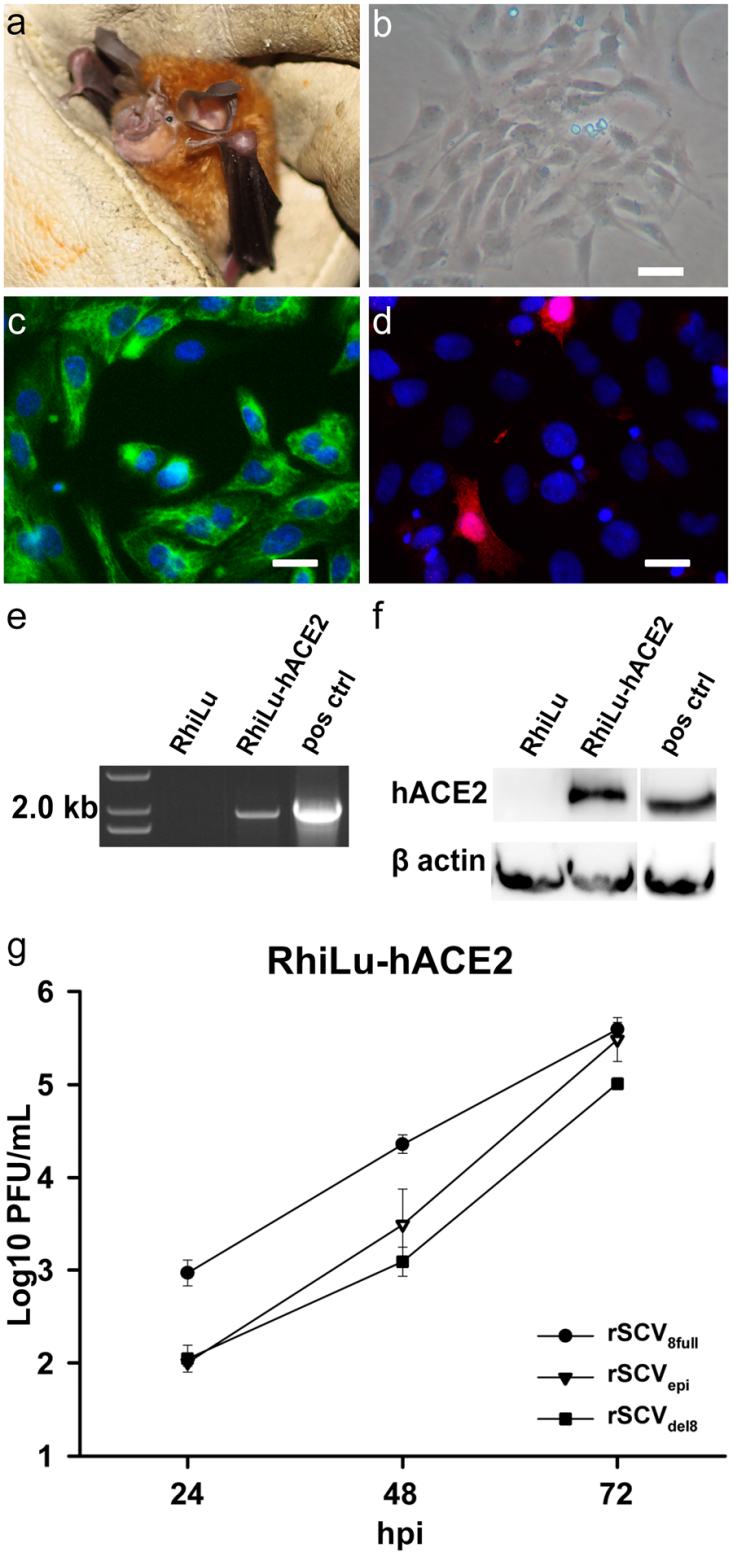
Generation of an hACE2-transgenic bat cell line for infection experiments with ORF8 variant rSCV. (**a**) Picture of an African *Rhinolophus alcyone* bat (copyright Victor Max Corman). Primary cell culture and immortalization of *R. alcyone* embryonic lung cells (**b**) was done as described in the Material and Methods section. To determine the RhiLu cell type the epithelial protein marker cytokeratin (**c**) and the fibroblast marker S-100A4 (**d**) were detected by immunofluorescence assay using mouse-anti cytokeratin or rabbit-anti S-100A4 Ig. Secondary detection was performed by incubating with goat-anti-mouse cyanin 2- or goat-anti-rabbit cyanin 3-labeled Igs. The bars represent 20 µm. (**e**) Integration of *hACE2* into the genome of RhiLu cells was verified by PCR. The vector containing the *hACE2*-puromycin resistance gene construct was used as a PCR positive control. (**f**) Expression of hACE2 was confirmed by Western blot analysis using mouse anti-hACE2 Ig (1:1,000). In addition, β actin protein was detected using rabbit anti-β-actin Ig (1:2,000) to ensure that similar protein amounts were applied. MA104 cells, expressing hACE2 naturally, served as positive control. (**g**) Virus replication of rSCV_8full_, rSCV_epi_ and rSCV_del8_ was observed by titration of supernatants sampled at 24, 48, and 72 hpi. Cells were infected in triplicates at an MOI of 0.001. Error bars represent standard deviation of the mean.

In previous studies, it has been suggested that the function of ORF8 may be more relevant for replication in cells from the actual animal reservoir, than for replication in human or primate cell cultures^30^. Because bats of the genus *Rhinolophus* are the *bona-fide* animal reservoir for SARS-related CoV^2^, an epithelial cell line was generated from the lung of *Rhinolophus alcyone* (Fig. 3a). One pregnant female individual was euthanized and embryonal tissues were prepared using techniques described previously (Fig. 3b)^31–33^. The resulting immortalized *Rhinolophus* lung cell culture, termed RhiLu, clearly expressed cytokeratin as a marker for epithelium (Fig. 3c), and hardly showed any expression of the fibroblast marker S-100A4 (Fig. 3d), suggesting that the cell culture mainly contains epithelial cells. Initial infection experiments in RhiLu cells identified these cells to be non-permissive for SARS-CoV infection. By use of VSV-G protein-pseudotyped lentiviral particles, RhiLu cells were therefore transduced with the gene for the SARS-CoV entry receptor, human angiotensin-converting enzyme 2 (hACE2), and selected to stably express hACE2 upon co-expression of a puromycin resistance gene from the same lentiviral vector. Chromosomal integration of the *hACE2* gene and expression of the hACE2 protein were verified by RT-PCR and Western blot analysis (Fig. 3e, f). The resulting *hACE2*-transgenic cells termed RhiLu-hACE2 were highly permissive for SARS-CoV infection.

RhiLu-ACE2 cells were infected with all three ORF8 virus variants at MOI = 0.001. Supernatants were harvested 24, 48 and 72 hpi and titered. As shown in Fig. 3g, the same hierarchy of viral replication levels as in VeroFM cells was observed (rSCV_8full_ > rSCV_epi_ > rSCV_del8_). These results did not point to a specific effect of the gene product of ORF8 on replication in bat cells but rather suggested a general promoting effect on virus replication conferred by ORF8.

### SARS-CoV ORF8 determines replication in non-host cell lines and in human airway epithelial cultures

**Figure 4:**
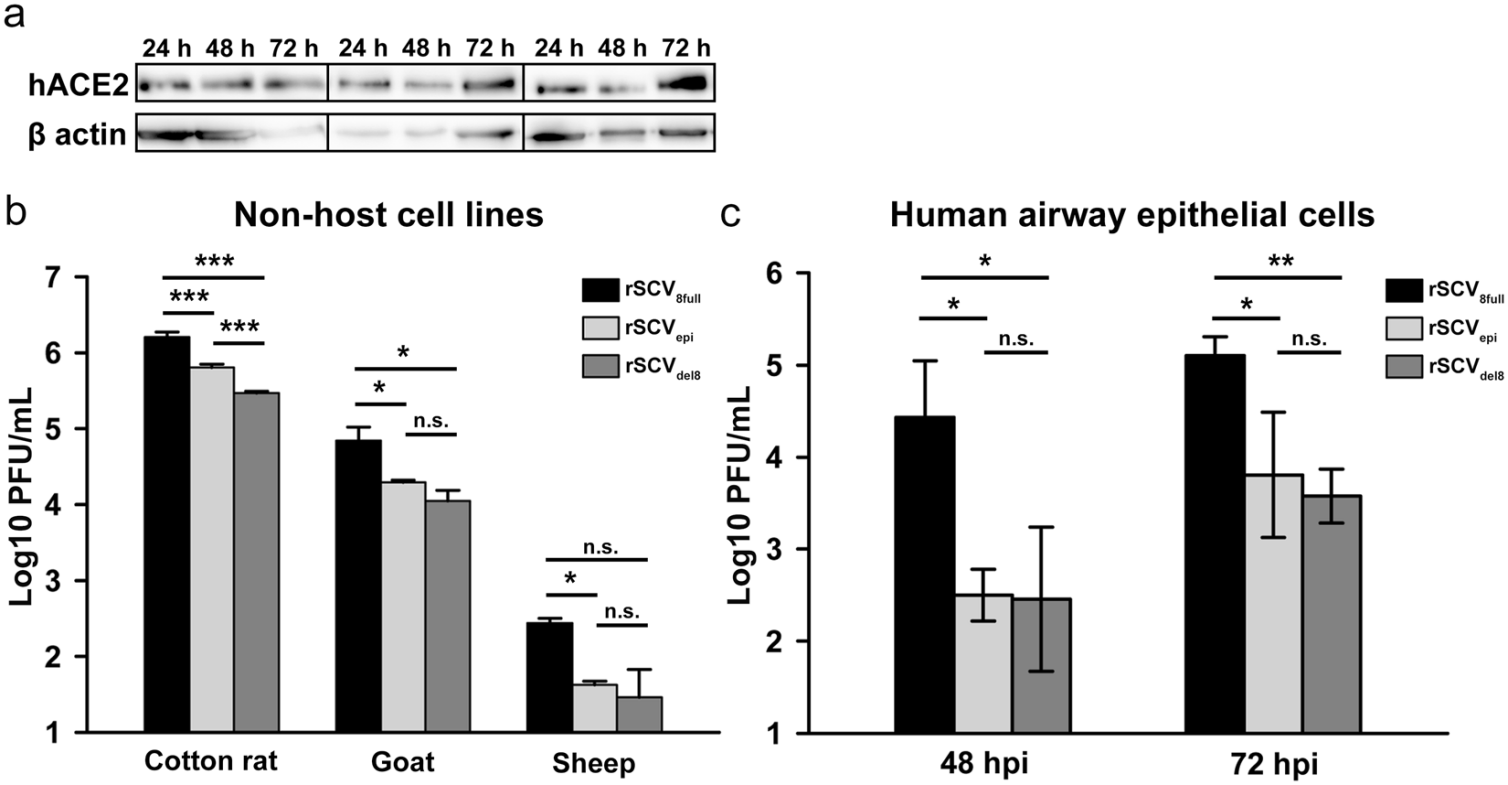
Replication of ORF8 variants in different “non-SARS-CoV-host” cell lines and differentiated human airway epithelial cells. (**a**) Cotton rat, goat, and sheep cells were transduced with lentiviruses to transiently express the SARS-CoV receptor hACE2 for at least 72 h. Expression of hACE2 was verified by Western Blot analysis, detection of β actin served as a loading control. To improve clarity blots were cropped. Full length blots are presented in Supplementary Fig. S1. (**b**) Cells were infected in triplicates with different ORF8 variants at MOI 0.001 24 h after lentiviral transduction, 24 hpi supernatants were sampled and virus replication determined by plaque titration. (**c**) Differentiated human airway epithelial cells were infected in triplicates at MOI = 0.1, supernatants were sampled at 48 and 72 hpi for plaque titration. The experiment was done twice. Shown is one representative experiment. Error bars represent standard deviation of the mean. Significance of replication differences between virus variants was determined by *t-Test* (***p ≤ 0.001, **p ≤ 0.001, *p ≤ 0.05, n.s., not significant p > 0.05).

To determine whether the effect was more general and independent of host taxa, a low-passage airway epithelial cell line from *Sigmodon hispidus* (cotton rat)^34^ and primary lung cell lines from *Capra hircus* (goat) and *Ovis aries* (sheep) were transduced using vesicular stromatitis virus G protein pseudotyped lentiviruses to transiently express hACE2 as described above (Fig. 4a), and subsequently infected with the ORF8 virus variants at MOI = 0.001. Again the same hierarchy of viral replication levels, namely rSCV_8full_ > rSCV_epi_ > rSCV_del8_, was observed at 24 hpi (Fig. 4b). Virus rSCV_8full_ replicated significantly less efficiently than rSCV_epi_ in all three “non-host” cell lines (*t*-Test: p = 0.001, p < 0.05, p < 0.01 for cotton rat, goat and sheep, respectively). Replication differences were levelled off by 48 hpi, when all viruses reached the plateau of replication. Of note, the utilized cell cultures in total represent hosts from four different orders of Euarchontoglires and Laurasiatheria, the two main groups of mammals.

As the virus variants constructed in this study corresponded to viruses from the three major phases of the human SARS epidemic, we were interested to see whether the deletions have an effect on human respiratory tract infection as represented by *in-vitro* differentiated human airway epithelial cultures (HAE). Infection was done at MOI = 0.01, and virus production was measured by plaque titration after 48 and 72 hpi in supernatants. At both time points rSCV_8full_ replicated to significantly higher titers than rSCV_epi_ or rSCV_del8_ in HAE (Fig. 4c).

## Discussion

Here we have shown by viral reverse genetics and advanced cell culture models that SARS-CoV ORF8 facilitates viral replication irrespective of the host cell system. These data suggest the occurrence of an attenuating mutation in the initial phase of the human SARS epidemic.

SARSr-CoV is a paradigmatic pathogen for the study of viral reservoirs and the processes involved in epidemic emergence^8,14,15,35^. The acquisition of certain spike proteins by recombination may have formed the viral lineage that emerged from the reservoir and established itself in humans during the SARS epidemic^6–8^. The gradual deletion of ORF8 constituted the most obvious change in SARS-CoV after emergence. Because few changes occurred in other parts of the genome, we have focused our study on ORF8 and kept the rest of the genome constant representing the sequence of SARS-CoV strain Frankfurt-1. This prototype virus was isolated from the late phase of the SARS-CoV epidemic when transmission chains already occurred in countries outside China^36^. Even in this late epidemic strain, a reconstitution of the full ORF8 reading frame led to an increased replicative capability, suggesting that the viral genome was still compatible with its primordial ORF8 element in spite of possible onward evolution in humans^9^.

Compared to earlier observations with ORF8-deleted SARS-CoV, our results show a clear phenotypic difference in replication in relevant models of human respiratory tract infection. The phenotypic difference depends on the presence of full ORF8, with enhanced replication as opposed to the deletion variant. Earlier experiments in which no clear differences in replication have been noted were aimed at the identification of strong attenuation markers as necessary for live vaccine development, and hence worked at very high MOIs at or above 1^22^. We have also included such high MOIs in our experiments for comparison, and our results were highly concordant with those earlier studies. For instance, like in the study by Yount *et al*.^22^, differences between viruses with full ORF8 and 29nt deletions were only about three-fold at MOI = 1. Natural infection, however, does not involve high virus doses^37^. When a virus is passed from human to human, the stochastic nature of infection success implicates that inocula just below or above one unit of human-infectious virus are transmitted. For instance, during influenza A transmission in ferrets as few as 2 virus units were transmitted between animals^38^. Under conditions of inter-host transmission, the observed phenotypic differences as observed in our study may have significant effects on viral fitness^37^.

In endemic viral infections, loss of fitness should cause viral lineage extinction in competition with more reproductively capable lineages that co-circulate within the host population^39^. After a single-time zoonotic introduction, however, competing viral lineages are unlikely to exist. Variants with slightly deleterious mutations, randomly selected through transmission bottlenecks, can continue to reproduce in spite of reduced fitness – an effect known as founder effect. Interestingly, it has been shown by *in-vitro* studies that virus populations with reduced initial fitness suffer less from slightly deleterious mutations than populations that replicate on peak fitness level^40^. Reduced initial fitness is a condition that can be expected in early-stage zoonotic epidemics when the virus is not yet adapted to the new host environment^41^.

Considering the present results, we therefore suggest that the 29 nt deletion in SARS-CoV is the result of a founder effect that has permitted survival in spite of reduction of fitness. This interpretation contrasts with the earlier notion that the 29 nt deletion reflects adaptation to humans. The conclusion of adaptation is partly based on results from expression of ORF8 or its truncation products in overexpression systems or expression in heterologous virus genomes, suggesting various influences on virus-cell interaction^16–21^. For instance, one study provided evidence for IFN evasion mediated by ORF8b^17^. This is not confirmed by our experiments studying ORF8 in full virus context. Other authors have proposed that the 29 nt deletion may have been neutral for fitness after viral host transition to humans, assuming that ORF8 may elicit a function that is only relevant in the bat host^9,22^. However, we have not observed any bat-specific effects in cell culture, and rather show that ORF8 optimizes fitness irrespective of the host cell system, including hosts that are irrelevant for the SARS-CoV chain of emergence^11^. Our experiments suggest that only the deletion of further portions after initial fragmentation of ORF8 may have been neutral to fitness. Such deletions were seen during very late phases of the SARS epidemic in Hong Kong^9^.

Deletions in accessory reading frames were also oberserved in MERS-CoV^23,24,42,43^. Transmission of deleted variants was confirmed in an outbreak in Jordan, involving deletions in ORF4a, ORF3 and potentially other parts of the genome^23,24^. The available studies leave it open whether these deletions involved changes of replication level or virulence. However, it is known that ORF4a acts as an effective antagonist of MDA5-dependent induction of type I IFN^44^, and that MERS-CoV is highly sensitive against type I IFN in human airway epithelial cultures^45^. Based on these known mechanisms, attenuation rather than human adaptation should be considered. Our results are also relevant in the context of a series of experimental studies recently conducted to understand potential adaptive changes in the 2014 Ebola virus Makona outbreak in West Africa. Whereas initial studies suggested human adaptation with increase of replication level during the outbreak^46–50^, later studies found that a late-outbreak strain rather caused reduced virulence and prolonged survival of experimental animals^51^. These results provide another reminder of the fact that outbreak-associated mutations do not have to increase replication or virulence.

It is interesting to consider the consequences for viral propagation conferred by a reduction of viral replication level. As pointed out in Marzi *et al*. for Ebola virus Makona, a prolonged survival time as seen with late epidemic strains could have increased the duration of infectious virus shedding in humans and may thus have increased the long-term fitness of those viruses^51^. We cannot, at present, exclude whether similar effects may have provided a fitness advantage to SARS-CoV on host population level, such as by keeping infected individuals socially interactive for prolonged times while infected with a slightly attenuated virus. On an individual level, however, a reduction of replication level is likely to attenuate the pathogenicity of infection and reduce the health burden caused by a given outbreak.

It may be seen as a weakness in our present study that no experimental animals were infected as in Marzi *et al*. However, mouse models of SARS-CoV do not reflect human disease as accurate as macaques do for Ebola virus, and the HAE culture system used in the present study already provides an appropriate model for the authentic site of replication of SARS-CoV in the human body (Ebola virus infection cannot be modeled by organ-specific cultures). Moreover, the infection phenotypes as seen in our study for ORF8_full_ and ORF8_epi_ have already been demonstrated in mice, and the corresponding effects in cell culture based on those variants have been reproduced in our study^22^. We have therefore avoided additional animal experimentation and focused on human epithelial models with low inoculation doses such as seen in natural infections.

Our data suggest that SARS-CoV has suffered an attenuating mutation by the 29 nt deletion that constitutes a landmark genetic change. The SARS epidemic in 2003 may have taken a more severe course if not involving this mutation. Further work, including work in experimental animals, will be required to understand and confirm whether the absence of ORF8 in European bat-associated SARSr-CoV correctly predicts lesser epidemic risks as compared to Asian strains.

## Materials and Methods

### Sample collection and processing

In total, 827 bats were sampled in four countries (Bulgaria, Italy, Slovenia, Spain). All animals were handled according to national and European legislation for the protection of animals (EU council directive 86/609/EEC). Licenses for sampling of bats using mist nets, hand nets or harp traps were obtained from the respective countries and authorities: Bulgarian Ministry of Environment and Water, permit No. 192/26.03.2009^13^, Italien Ministry of the Environment, permit No. 192/26.03.2009^52^, Slovenian Environment Agency, permit No. 35701-80/2004^53^, Service for the Biodiversity Conservation of the Rural Counseling of the Xunta de Galicia, Spain, permit No. 52/2010 n.s. 13697. No animals were sacrificed during this study. All animal handling and sampling was done by trained personnel, with animal safety and comfort as the first priority during minimally invasive sampling (collection of faeces). Bat species were identified on site and, if necessary, mitochondrial DNA in representative fecal samples was amplified and sequenced for species confirmation as described previously^54^. Captured bats were freed from nets immediately and put into cotton bags for 2 to 15 min to allow them to calm down before examination. While being kept in bags, bats produced fecal pellets that were transferred to 500 µl RNAlater RNA stabilization solution (Qiagen, Hilden, Germany) for sample processing. After homogenization, 50 µl of the suspension was resuspended in 560 µl of buffer AVL from the Qiagen viral RNA minikit and processed according to manufacturers instructions. The elution volume was 50 µl.

### General cell culture procedures

Cells were grown in Dulbecco’s Modified Eagles Medium (DMEM) as described earlier^31^. For titration of rSCV Vero E6 cells (ATCC CRL-1586) were used. For infection studies African green monkey kidney cells, VeroFM (kindly provided by Jindrich Cinatl, University of Frankfurt) and MA104 (a gift from Friedemann Weber, University of Marburg), *Rhinolophus alcyone (R.alcyone)* embryonic lung cells (RhiLu, prepared in-house as described below), as well as bronchial epithelial cells from *Sigmodon hispidus* (cotton rat) and lung cells from *Capra hircus* (domestic goat) and *Ovis aries* (domestic sheep) were used^55,56^. HAE cultures were generated and cultured as described elsewhere^57^.

### Generation and characterization of *R. alcyone* lung cell cultures

Bats were caught in Ghana under research permit no. CHRPE49/09; A04957 Wildlife Division, Forestry Commission, Accra, Ghana. Primary bat cell culture and immortalization of RhiLu cells by lentiviral transduction of the simian virus 40 large T antigen and genotyping were done as previously described^31,33^. To determine the RhiLu cell type the epithelial protein marker cytokeratin and the fibroblast marker S-100A4 (calcium binding protein A4 or fibroblast specific protein 1) were stained by immunofluorescence assay using mouse anti-cytokeratin (ab7753) or rabbit anti-S-100A4 immunoglobulins (Ig, ab27957; both supplied by abcam, Cambridge, UK). Secondary detection was performed by incubation with goat anti-mouse cyanin 2- or goat anti-rabbit cyanin 3-labeled Igs.

### Generation of a SARS-CoV susceptible bat cell line by lentiviral transduction

Since RhiLu cells were not susceptible to SARS-CoV the receptor hACE2 was stably transfected by lentiviral transduction^58^. Genomic integration of *hACE* was verified by PCR on genomic DNA. PCR was performed using a *hACE2* gene specific forward primer (GAATGTAAGGCCACTGCTCAACTA) and a puromycin resistance gene specific reverse primer (TCAGGCACCGGGCTTGC) yielding a 1.9 kb amplicon. Expression of hACE2 was confirmed by Western blot analysis as described earlier^58,59^. Protein lysates were separated on a 10% SDS-PAGE gel, and blotted onto a 0.45 µm polyvinylidene fluoride membrane. Primary detection of hACE2 was done using a mouse anti-hACE2 Ig (1:1,000; R&D Systems, Wiesbaden-Nordenstadt, Germany), for secondary detection a goat anti-mouse horseradish peroxidase (HRP)-conjugated Ig (1:20,000) and SuperSignal^®^ West Femto Chemiluminescence Substrate (Fisher Scientific, Schwerte, Germany) were used^60^. As a loading control samples were analyzed for β actin expression with a rabbit anti-βactin Ig (1:2,000; Sigma-Aldrich, Munich, Germany) and a goat anti-rabbit horseradish peroxidase-labeled Ig (1:20,000)^61^.

### Generation of recombinant SARS-CoV

Recombinant SCV was generated as previously described^25^. The single full-length ORF8 was generated by inserting 29 nts into ORF8a/b. In an overlap extension PCR two templates, generated by using primers F26020F (CGGCTCTTCAGGAGTTGCTA) in combination with 29nt-rev (TCCATTCAGGTTGGTAACCAGTAGGACAAGGATCTTCAAGCACATGA) and 29nt-fwd (CTGGTTACCAACCTGAATGGAATATAAGGTACAACACTAGGGGTAATACT) with pB-fwd (GCCCTTAAACGCCTGGTTGCTAC), were fused. Underlined nts indicate overlapping regions leading to the introduction of 29 nts. The PCR product was inserted into subclone pEF via restriction sites BamHI and NotI. ORF8 was deleted from subclone pDEF by Phusion^®^ Site-Directed Mutagenesis (Fisher Scientific) using 5′-phosphorylated primers delO8-1-fwd (AATGTCTGATAATGGACCCCAATCAAACCAACGTAGTGC) and delO8-1-rev (GTTCGTTTAGACTTTGGTACAAGGTTCTTCTAGATCC) or delO8-2-fwd (TAAAATGTCTGATAATGGACCCCAATCAAACCAACG) and delO8-2-rev (TTGTTCGTTTAGACTTTGGTACAAGGTTCTTCTAGATCC). Underlined nts are the substitutional sequence for ORF8 compared to delO8-1. Assembly of full-length SARS-CoV genome plasmids and rescue of recombinant viruses were done as described before^25^. Briefly, full-length SARS-CoV plasmid was linearized by NotI and *in-vitro* transcribed (mMESSAGE mMACHINE® Kit, Applied Biosystems, Darmstadt, Germany). Capped mRNA was electroporated into baby hamster kidney cells and supernatant was subsequently transferred to susceptible VeroFM cell culture 24 h post electroporation. Recombinant virus was harvested three days post infection. The ORF8 mutations were verified by PCR using a specific reverse primer F29260R (TTTGTATGCGTCAATGTGCTTG) for reverse transcription and ORF8 covering forward primer F27626F (GAGAAAGACAGAATGAATGAGC) and reverse primer F28182R (GGGTCCACCAAATGTAATGCGG) for conventional PCR. Sequencing of the PCR product ensured the integrity of the introduced mutations. Expression of the nucleocpasid was confirmed by Western blot analysis as described above. Primary detection was done using a rabbit anti-nucleocapsid Ig (1: 500; abcam). For secondary detection a goat anti-rabbit HRP-conjugated Ig (1:20,000) and and SuperSignal^®^ West Femto Chemiluminescence Substrate (Fisher Scientific) was used.

### Generation of transiently hACE2 expressing cell lines

Cells were seeded according to their size and growth rate to yield 80% confluence in 24-well plates. After attachment overnight, cells were infected with lentiviruses to yield 50 ng reverse transcriptase activity per well in a reduced cultivation volume of 200 µL DMEM per well for at least 24 h. Expression levels of hACE2 were determined by Western blot analysis as described above in a time course experiment. Protein expression levels were constant for three days after transduction. Therefore, cells were infected with rSCV 24 h post transduction as described below.

### Virus infection

Cells (4 × 10^5^ cells/mL) were seeded in 24-well plates and if necessary pre-incubated with 200 µl recombinant universal type I IFN alpha (PBL InterferonSource, Piscataway, USA) for 16 h prior to infection. Infections with rSCV_8full_, rSCV_epi_ and rSCV_del8_ were done at an MOI ranging from 0.001 to 1. Virus was diluted in OptiPRO^TM^ serum-free medium or in HBSS buffer in case of HAE cultures. Cells were inoculated for 1 to 2 h at 37 °C, washed twice with PBS or HBSS, and supplied with fresh DMEM or HAE cell culture medium^57^. Supernatants were taken at designated time points usually 8, 24, 48, and 72 h post infection and stored at −70 °C for titration or real-time RT-PCR analysis. All virus containing samples were mixed with equal volumes of 0.5% gelatin in OptiPRO^TM^ (stock solution 5% gelatin in water) for stabilization of infectious particles. All infection experiments were done under biosafetly level 3 conditions with enhanced respiratory personal protection equipment.

### Plaque titration

Titration of rSCV was done as previously described^25,62^. Vero E6 cells (3.5 × 10^5^ cells/mL) were infected with a serial dilution (in OptiPRO^TM^) of virus infected cell culture supernatants for 1 h at 37 °C. After removing the inoculum cells were overlaid with 2.4% Avicel (FMC BioPolymers, Brussels, Belgium) 1:2 diluted in 2 × DMEM supplemented with 2% Penicillin/Streptomycin, 2% L-glutamine, 2% non-essential amino acids, 2% sodium pyruvate and 20% fetal bovine serum. Three days after infection the overlay was discarded, cells were fixed in 6% formaldehyde and stained with a 0.2% crystal violet, 2% ethanol and 10% formaldehyde containing solution.

The datasets generated during and/or analysed during the current study are available from the corresponding author on reasonable request.

## Electronic supplementary material

Dataset 1
