# Supplementary material for: Attenuation of replication by a 29 nucleotide deletion in SARS-coronavirus acquired during the early stages of human-to-human transmission
Source: Sci Rep. 2018 Oct 11;8:15177. doi: 10.1038/s41598-018-33487-8 (PMC6181990; doi:10.1038/s41598-018-33487-8)
Supplement: Supplementary file 1 — Dataset 1 [file 41598_2018_33487_MOESM1_ESM.pdf]

# Attenuation of replication by a 29 nucleotide deletion in SARS-coronavirus acquired during the early stages of human-to-human transmission

Doreen Muth[1,2], Victor Max Corman[1,2], Hanna Roth[2], Tabea Binger[2], Ronald Dijkman[3,4], Lina Theresa Gottula[1,2], Florian Gloza-Rausch[5], Andrea Balboni[6], Mara Battilani[6], Danijela Rihtarič[7], Ivan Toplak[7], Ramón Seage Ameneiros[8,9], Alexander Pfeifer[10], Volker Thiel[3,4], Jan Felix Drexler[1,2], Marcel Alexander Müller[1,2], Christian Drosten[1,2*]

[1]Charité-Universitätsmedizin Berlin, corporate member of Freie Universität Berlin, Humboldt-Universität zu Berlin, and Berlin Institute of Health, Institute of Virology, Charitéplatz 1, 10117 Berlin, Germany; German Center for Infection Research (DZIF), Berlin, Germany

[2]Institute of Virology, University of Bonn Medical Centre, Sigmund-Freud-Str. 25, 53127 Bonn, Germany

[3]Federal Department of Home Affairs, Institute of Virology and Immunology IVI, Bern and Mittelhäusern, Sensemattstrasse 293, 3147 Mittelhäusern, Switzerland

[4]Department of Infectious Diseases and Pathobiology, Vetsuisse Faculty, University of Bern, Länggassstrasse 122, 3012 BernSwitzerland

[5]Noctalis, Centre for Bat Protection and Information, Oberbergstraße 27, 23795 Bad Segeberg, Germany

[6]Dipartimento di Scienze Mediche Veterinarie, Facoltà di Medicina Veterinaria, Alma Mater Studiorum-Università di Bologna, Via Tolara di Sopra 50, 40064 Ozzano Emilia (BO), Italy

[7]Virology Unit, Institute of Microbiology and Parasitology, Veterinary Faculty, University of Ljubljana, Gerbičeva 60, 1000 Ljubljana, Slovenia

[8] Institute of Evolutionary Ecology and Conservation Genomics, University of Ulm, Albert-Einstein Allee 11,89069 Ulm, Germany

[9]Group Morcegos de Galicia, Drosera Society, Pdo. Magdalena, G-2, 2º esq, 15320 As Pontes, Spain

[10]Institute for Pharmacology and Toxicology, University of Bonn, Sigmund-Freud-Str. 25, 53127 Bonn, Germany

*Corresponding author: Christian Drosten, e-mail: christian.drosten@charite.de, tel: +49-30-450525091, fax: +49-30-450 752 5907

Word count: 3495

**Supplementary Fig. S1: Expression of hACE2 after transduction of "non-SARS-CoV-host" cell lines.** Cells from sheep, cotton rat and goat were seed in 24-well plates to reach 80% confluency for infection with lentiviruses (total of 50 ng reverse transcriptase activity) to induce transient expression of hACE2. Protein expression was analysed at 24, 48 and 72 h post infection. HACE2 (ca. 140 kDa) was detected using anti-hACE2 Ig (1:1,000; R&D Systems, Wiesbaden-Nordenstadt, Germany) and a goat anti-mouse horseradish peroxidase (HRP)-conjugated Ig (1:20,000) (in a) lanes 4-9, in b) lane 4-6). The expression of β-actin (ca. 42 kDa) served as a loading control using a rabbit anti-β-actin Ig (1:2,000; Sigma-Aldrich, Munich, Germany) and a goat anti-rabbit horseradish peroxidase-labeled Ig (1:20,000) (lanes 4-9 in c) and lanes 4-6 in d). Lanes 1-3 of a), b), c) and d) carry samples non-relevant for the publication. Chemiluminscence signals were detected using SuperSignal® West Femto Chemiluminescence Substrate (Fisher Scientific, Schwerte, Germany) after 1 min (a), 10 sec (b), 10 sec (c) and 1 min (d) exposure time in a FusionFX7 (PeqLab/Vilbert Lourmat, Erlangen, Germany). HACE2 signals were visible during actin detection because Western Blots were not stripped between incubation with different primary antibodies. M, PageRuler Prestained Protein Ladder (Fisher Scientific).

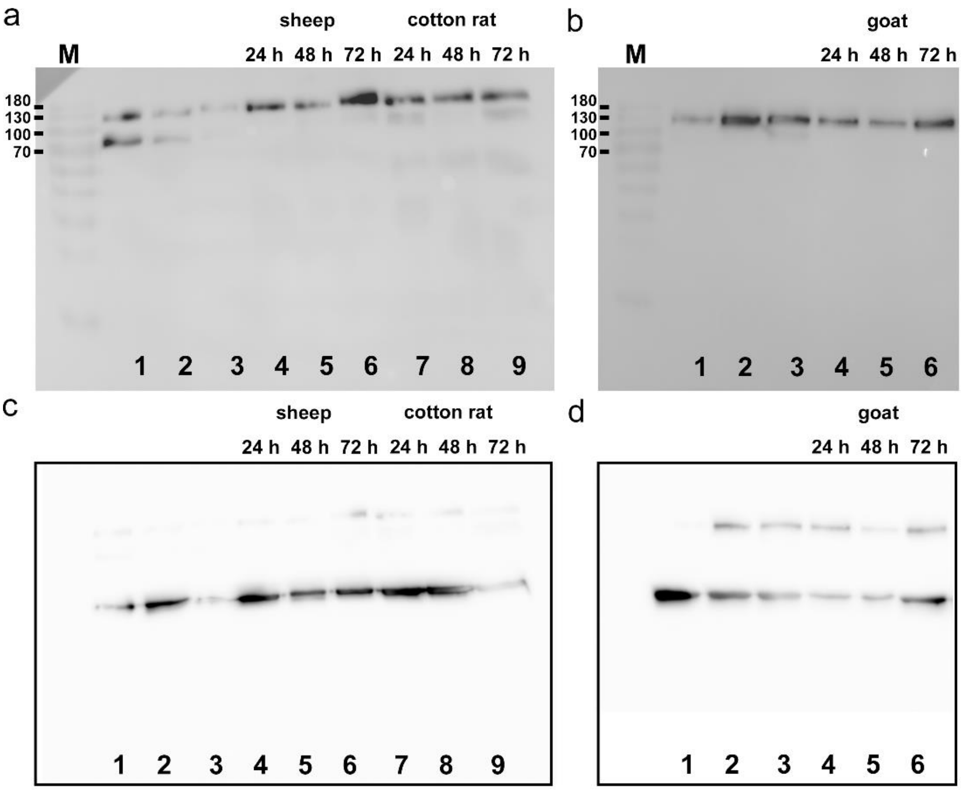
a
sheep
cotton rat
M
24 h 48 h 72 h 24 h 48 h 72 h
180
130
100
70
1 2 3 4 5 6 7 8 9
b
goat
M
24 h 48 h 72 h
180
130
100
70
1 2 3 4 5 6
c
sheep
cotton rat
24 h 48 h 72 h 24 h 48 h 72 h
1 2 3 4 5 6 7 8 9
d
goat
24 h 48 h 72 h
1 2 3 4 5 6
